# RWA-BFT: Reputation-Weighted Asynchronous BFT for Large-Scale IoT

**DOI:** 10.3390/s25020413

**Published:** 2025-01-12

**Authors:** Guanwei Jia, Zhaoyu Shen, Hongye Sun, Jingbo Xin, Dongyu Wang

**Affiliations:** 1 Shuohuang Railway Development Co., Ltd., National Energy Group, Cangzhou 062356, China; 11110575@ceic.com (G.J.); 11111156@ceic.com (J.X.); 2 School of Artificial Intelligence, Beijing University of Posts and Telecommunications, Beijing 100876, China; zhaoyushen@bupt.edu.cn (Z.S.); shy18501176753@bupt.edu.cn (H.S.)

**Keywords:** blockchain, IoT, BFT, asynchronous consensus, scalability

## Abstract

This paper introduces RWA-BFT, a reputation-weighted asynchronous Byzantine Fault Tolerance (BFT) consensus algorithm designed to address the scalability and performance challenges of blockchain systems in large-scale IoT scenarios. Traditional centralized IoT architectures often face issues such as single points of failure and insufficient reliability, while blockchain, with its decentralized and tamper-resistant properties, offers a promising solution. However, existing blockchain consensus mechanisms struggle to meet the high throughput, low latency, and scalability demands of IoT applications. To address these limitations, RWA-BFT adopts a two-layer blockchain architecture; the first layer leverages reputation-based filtering to reduce computational complexity by excluding low-reputation nodes, while the second layer employs an asynchronous consensus mechanism to ensure efficient and secure communication among high-reputation nodes, even under network delays. This dual-layer design significantly improves performance, achieving higher throughput, lower latency, and enhanced scalability, while maintaining strong fault tolerance even in the presence of a substantial proportion of malicious nodes. Experimental results demonstrate that RWA-BFT outperforms HB-BFT and PBFT algorithms, making it a scalable and secure blockchain solution for decentralized IoT applications.

## 1. Introduction

### 1.1. Background

IoT technology acts as a bridge between the physical and digital worlds and has developed rapidly in recent years. According to IDC, by 2025, more than 41 billion IoT devices will be deployed globally, and the market size is projected to exceed $1.2 trillion [1]. IoT devices are extensively used in various domains, such as smart homes, smart manufacturing, smart cities, and healthcare monitoring. In smart cities, IoT devices collect and analyze critical data, including traffic flow, environmental monitoring, public safety, and infrastructure conditions. For instance, sensors embedded in streetlights and traffic signals provide city managers with real-time operational data. This allows for improved resource allocation and faster emergency response [2].

The rapid growth of IoT has also introduced challenges related to data security and privacy protection. IoT devices and systems are becoming increasingly susceptible to cyber-attacks, particularly during data transmission and storage, where security vulnerabilities are more likely to arise [3,4]. IoT data processing often depends on centralized cloud platforms. This approach increases operational and maintenance costs and introduces risks, such as single points of failure in centralized systems. A failure in any central server can disrupt the entire network. This poses an unacceptable risk, especially in the management of critical infrastructure [5]. As the number of devices increases, existing data processing architectures face significant challenges in scalability and efficiency. Managing the rapidly growing data streams and expanding device networks has become a critical issue requiring immediate attention.

Blockchain technology, as a distributed ledger, verifies and records transactions across multiple network nodes, ensuring both data integrity and security. Each transaction is stored in a public, immutable block, which is cryptographically linked into a continuous chain. This architecture ensures data transparency and immutability, significantly enhancing trust and security within the network [6].

The core of blockchain technology lies in its decentralized nature, which removes the need to trust a central server or administrator, thereby reducing the risks of tampering or attacks. Furthermore, blockchain maintains data consistency across all nodes in the network using consensus mechanisms such as Proof of Work (PoW) and Proof of Stake (PoS) [7]. Smart contracts represent another critical innovation in blockchain technology. These self-executing protocols operate on the blockchain, enabling the enforcement of contract terms without the need for third-party intervention. This feature enables automated transactions and applications, greatly expanding the range of blockchain technology’s potential applications [8]. Despite the many potential advantages of blockchain technology, its practical applications continue to encounter scalability and performance bottlenecks. Current blockchain networks have limited capacity to handle large-scale transactions and rapid data transmission. These challenges require technological innovation and protocol optimization for effective resolution [9]. 

The application of blockchain technology to large-scale IoT opens up new possibilities for addressing various challenges in the IoT domain. The decentralized nature of blockchain reduces IoT devices' dependence on central servers, thereby lowering the risk of network paralysis caused by central server failures. Moreover, the immutability of blockchain significantly enhances data security and transparency. This feature is particularly critical for IoT applications that demand high levels of trust, such as medical devices and transportation systems. Blockchain technology allows IoT devices to exchange data securely within a decentralized environment, removing the need for centralized data processing centers. This peer-to-peer communication model improves data processing efficiency while also reducing security risks during data transmission [10]. Smart contracts on the blockchain can automate interactions between devices, including automatic payments, device maintenance, and energy management. These functions are essential for achieving IoT device automation [11].

### 1.2. Difficulties

A core advantage of blockchain technology is its decentralized nature, which ensures system stability even if individual nodes are attacked or fail. However, decentralization also introduces performance challenges, particularly in handling large-scale transactions. In traditional blockchain systems, such as Bitcoin or Ethereum, every node must process and verify all transactions. This leads to significant delays and throughput limitations during periods of high transaction volume. For example, the Bitcoin network, due to design limitations, can process only seven transactions per second, which is insufficient to meet the demands of global financial transactions. While Ethereum has enhanced its functionality with smart contracts, it has faced severe network congestion when managing large-scale applications, such as CryptoKitties.

Applying blockchain technology to large-scale IoT scenarios presents a major challenge: effectively processing the massive data generated by numerous devices without compromising security or decentralization. The widespread distribution of IoT devices and their rapid data generation require blockchain systems to handle large transaction volumes efficiently and with low latency. This creates significant challenges for current blockchain scalability. Blockchain scalability refers to the system’s ability to meet new demands, such as higher transaction throughput, reduced node storage requirements, faster transmission rates, and improved consensus efficiency, particularly as large-scale IoT applications continue to grow [12]. The existing single-chain structure struggles to meet the data throughput demands of industrial parallel processing applications. Current multi-chain solutions face issues like centralization, limited application scenarios, and the inability to enable interaction between multiple chains [13].

Moreover, the transaction processing speed of blockchain directly affects its efficiency in scenarios requiring real-time data processing. The size of each block limits the number of transactions it can include, a constraint that becomes especially apparent in data-intensive IoT applications. As transaction volumes grow, blockchain networks become increasingly prone to congestion, leading to longer transaction confirmation times. In IoT scenarios, frequent exchanges of small data packets between devices are significantly affected, severely reducing system responsiveness and overall usability.

To improve blockchain's processing capacity, several solutions have been proposed, including increasing block size or adjusting consensus mechanisms to enhance efficiency. However, these measures could undermine blockchain security, increasing the risk of system attacks or data tampering. Although blockchain transparency can enhance user trust, it may also lead to the exposure of sensitive data.

To address the scalability challenges encountered by the aforementioned blockchain in IoT scenarios, this paper introduces an asynchronous BFT consensus algorithm leveraging reputation weight, referred to as RWA-BFT. The primary contributions of this work are outlined as follows:

1. To address the issue of limited scalability in existing IoT architectures integrated with blockchain in asynchronous network environments, this paper proposes a two-layer blockchain-based model. This model facilitates fine-grained transactions in the IoT, enabling seamless integration of the IoT device layer with the blockchain network layer.
2. This paper proposes a reputation-weighted asynchronous BFT consensus algorithm. The algorithm's core idea is to assess the reputation of nodes across the network, elect consensus nodes through voting, group them evenly, and monitor their behavior. It adopts intra-group consensus within groups and global consensus among master nodes to lower the communication complexity of the traditional PBFT algorithm, thereby enhancing consensus efficiency. The first layer (device layer) uses a reputation mechanism to exclude malicious IoT nodes from the current consensus process. The second layer (network layer) facilitates consensus among departmental entity nodes, ensuring secure IoT data interactions.
3. To simulate the asynchronous network environment of the Internet of Things, the proportion of malicious nodes was increased, and random delays were introduced. A comparative experiment was then conducted with two baseline algorithms, PBFT and HB-BFT, to validate the proposed algorithm’s ability to improve throughput, reduce delays, and maintain robustness in IoT scenarios.

## 2. Related Work

This paper introduces a highly scalable hybrid two-layer Byzantine Fault Tolerance (BFT) consensus algorithm tailored for IoT blockchain applications. This section provides an overview of the related technologies utilized in the proposed solution, focusing on blockchain and consensus algorithms.

### 2.1. Blockchain Characteristics

Blockchain is fundamentally a public, distributed database that maintains an ever-expanding encrypted ledger. From a data perspective, data recorded on the blockchain are immutable and cannot be altered. Unlike traditional databases that store all records on central servers, blockchain’s decentralized architecture ensures that each network participant retains a copy of all records. Technically, blockchain is an innovative internet-based system built on a peer-to-peer (P2P) network. The chain structure enables data verification and storage, the consensus mechanism facilitates data generation and updating, cryptographic algorithms ensure secure transmission and access, and smart contracts automate execution and control of data operations. As a result, blockchain is defined by its key characteristics: decentralization, immutability, traceability, and transparency.

**Figure 1:**
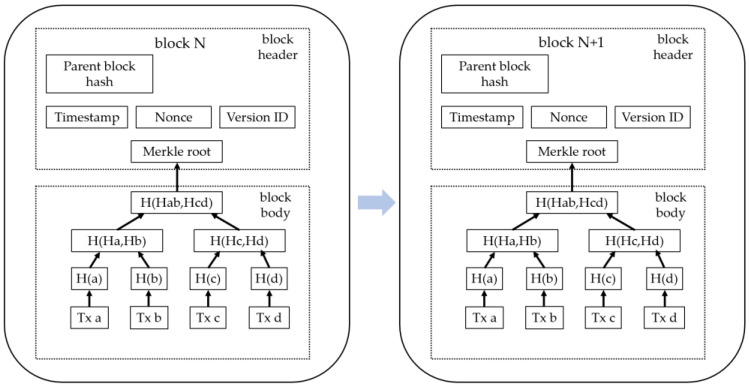
Schematic diagram of the block structure.

The schematic diagram of the block structure is shown in Figure 1. A block is a data structure containing historical transaction records, with the first block, called the genesis block, being the foundation of the blockchain. Each block includes a block header and a block body. The block header is 80 bytes in size and contains six fields: parent block hash, version number, timestamp, difficulty, nonce, and Merkle root. Each consecutive block includes the hash of the previous block. Hashes are irreversible, meaning a hash can be computed from a specific input, but the input cannot be reverse-engineered from the hash. Additionally, hashes are collision-resistant, meaning it is extremely unlikely for two different inputs to produce the same hash.

The integration of hashes into blockchain technology ensures strong security. Even the slightest modification to a block causes a significant change in its hash, which then propagates to all subsequent blocks. This feature makes it possible to detect any form of malicious tampering. The inclusion of hashes and timestamps ensures that blockchain ledger data is both secure and immutable. Furthermore, as transactions form the core components of a block, blockchain technology has been widely adopted in the financial sector.

The key characteristics of blockchain include the following:

- **Decentralization:** Decentralization is the core characteristic of blockchain. In a blockchain system, all decisions and consensus are achieved through the participation of all nodes. Each node in the system holds equal importance and maintains an identical ledger, recording the same transaction information. Any qualifying node can join or leave the blockchain system freely and swiftly, without requiring approval from a central authority.
- **Immutability:** Since blockchain is decentralized, each node retains an identical copy of the ledger. If a malicious node attempts to tamper with its ledger, it cannot alter the records stored on other nodes, rendering its local modifications ineffective. This is one of the reasons why blockchain ensures immutability. Another reason for immutability is that each block's header contains the hash value of the previous block and the Merkle root, derived from all transactions within the block. For a malicious node to alter a transaction in a block, it must ensure that the altered transactions still pass the Merkle tree verification, which, due to the collision resistance of hash functions, is nearly impossible. Similarly, if a malicious node attempts to construct a “legitimate” block to replace an existing one, it would need to generate a hash value for the new block that matches the original block’s hash value. Similarly, the collision resistance of hash functions ensures the impossibility of replacing an entire block.
- **Transparency:** Because blockchain is decentralized, every node retains an identical copy of the ledger, allowing each node to access the same information. Nodes can freely join or leave the blockchain system, and all nodes within the system can verify the transaction records, making the ledger public and transparent to anyone. Although this results in substantial redundancy, transparency is a crucial characteristic of blockchain and is of paramount importance in specific application scenarios.

### 2.2. BFT Consensus

Because blockchain systems are decentralized, with nodes distributed throughout the network, a comprehensive framework is urgently needed to maintain the system's orderly and fair operation, unify the blockchain version, and administer appropriate rewards or penalties to honest and malicious nodes, respectively. This framework necessitates a mechanism to establish proof. For example, if an honest node successfully competes for the right to record transactions on the blockchain ledger, it can receive a reward for creating a new block. Conversely, if a malicious node attempts to disrupt the network, it will be penalized. This mechanism is referred to as the consensus mechanism in a blockchain system. The purpose of a consensus protocol is to enable distributed nodes to agree on a specific piece of information or transaction. These protocols can be further subdivided into broadcast protocols and consensus protocols based on their application scenarios and implementation objectives.

First, the three network models of the blockchain consensus layer are introduced, which are categorized as synchronous, semi-synchronous, and asynchronous, based on different assumptions about network latency.

- **Synchronous Network Model:** In this model, the transmission time between any two nodes is smaller than a known fixed value, *d*. Algorithms like Paxos [14] and Raft [15] operate in this model.
- **Semi-Synchronous Network Model:** In this model, the message delivery time between any two nodes is not fixed and can vary over time, but its variation remains within polynomial bounds. The classic PBFT [16] algorithm operates under this model.
- **Asynchronous Network Model:** In this model, an adversary may arbitrarily alter the order of messages and introduce any delays in message transmission. In this system, the delivery time and order of messages are unpredictable.

The asynchronous network model requires the fewest assumptions and involves the most powerful adversaries among the three models. Consensus algorithms that work under the asynchronous network model are guaranteed to work under synchronous and semi-synchronous models, but the reverse does not hold.

#### 2.2.1. Synchronous and Semi-Synchronous Protocols

Semi-synchronous consensus protocols have gained significant attention for their high performance, driving the development of increasingly efficient algorithms. In the history of distributed systems, research on synchronous and semi-synchronous consensus protocols has reached a more mature stage.

In the domain of synchronous consensus protocols, Abraham et al. recently introduced Sync HotStuff [17], a high-performance protocol demonstrating that synchronous consensus can achieve functionality nearly equivalent to crash fault-tolerant protocols (CFT) like Paxos. In the semi-synchronous protocol domain, SiSi Duan et al. proposed the ByzID [18], leveraging intrusion detection systems, while Jian Liu et al. introduced FastBFT [19], which uses trusted hardware to improve both resilience and performance. Aublin et al. proposed the Aliph [20], Bahsoun introduced Adapt [21], and SiSi Duan developed the Cost [22], all designed to balance system performance and security through subprotocol switching. Amir introduced the Steward [23], which enhances system scalability through partitioning. In recent years, several practical semi-synchronous protocols have been introduced. For example, Yin et al. proposed HotStuff [24], and Golan-Gueta et al. introduced SBFT [25], both aiming to enhance system performance by reducing message complexity. These protocols have seen industrial adoption; HotStuff powers Facebook’s Libra blockchain, while SBFT supports VMware’s Concord blockchain.

#### 2.2.2. Asynchronous Protocols

Asynchronous consensus protocols have gradually transitioned from theory to practice in recent years. However, compared to synchronous and semi-synchronous protocols, asynchronous protocols remain less mature in both scientific research and practical applications. In practical applications, most blockchain systems in the industry rely on semi-synchronous consensus protocols, such as PBFT [16] and Tendermint [26]. This preference arises primarily from the high message complexity of asynchronous consensus protocols, which results in lower performance. Moreover, research on practical asynchronous algorithms remains less developed compared to semi-synchronous algorithms, limiting their widespread adoption.

The HoneyBadgerBFT [27], introduced in 2016, demonstrated that asynchronous consensus can achieve a throughput of 10,000 transactions per second. Several asynchronous protocols have been proposed in subsequent years, including BEAT [28], Dumbo [29], and EPIC [30]. These studies have laid a solid foundation for applying asynchronous consensus protocols in blockchain. For instance, Ant Chain has proposed a scheme using HoneyBadgerBFT as its consensus module.

Asynchronous consensus protocols have a rich history of “common” frameworks designed to build secure protocols through various approaches. The Asynchronous Common Subset (ACS) framework is a classical protocol framework for asynchronous Byzantine consensus protocols, which realizes atomic broadcasting in distributed systems. This is the core nature of the blockchain. The ACS framework, introduced by Ben-Or et al. in 1994 [31], has been applied in numerous subsequent works, including CKPSB [32] (Cachin et al., 2001), SINTRA [33] (Cachin et al., 2001), HoneyBadgerBFT [27] (Miller et al., 2016), BEAT [28] (Duan SiSi et al., 2018), Dumbo [29] (Guo et al., 2020), and EPIC [30] (Liu Chao and Duan SiSi et al., 2020).

ACS is widely regarded as the most practical framework for asynchronous consensus protocols. The primary distinction among protocols within this framework lies in their use of different modules to achieve varying levels of optimization. For example, HoneyBadgerBFT employs Erasure-Coding-based RBC for communication complexity optimization, BEAT introduces an FPCC-based scheme for further optimization, and EPIC incorporates Adaptive Security (AS) for enhanced communication efficiency. The Dumbo protocol optimizes the ACS framework by reducing the number of ABA instances to a constant, thereby improving system throughput and scalability. Beyond the ACS framework, other asynchronous consensus protocol frameworks exist, such as RITAS [34] (Moniz et al., 2011) and the asynchronous framework by Abraham [35] et al. (2019).

Synchronous or semi-synchronous Byzantine consensus protocols often integrate broadcast and consensus processes into an inseparable whole, making it difficult to distinguish between the two. In contrast, most asynchronous Byzantine consensus protocols can be clearly divided into two components: the Byzantine broadcast protocol and the Byzantine consensus protocol.

Byzantine Broadcast was first introduced in the Byzantine Generals Problem, which describes a scenario involving an army of multiple Byzantine generals who must decide collectively whether to attack or retreat, despite the presence of traitors among them. In this problem, a designated command sender issues orders to different generals. The command sender broadcasts messages in a manner that ensures all loyal generals reach a consensus decision, even in the presence of traitors. The Byzantine consensus scenario differs in that it lacks a designated command sender. Instead, the goal is for all generals to independently agree on and execute consistent orders.

#### 2.2.3. Byzantine Broadcast Protocol

This subsection describes two widely used Byzantine broadcast protocols: Reliable Broadcast (RBC) and Consistent Broadcast (CBC). The RBC protocol is more secure and ensures validity, consistency, correctness, and integrity of broadcasts. However, it does not guarantee the orderliness of broadcast messages. In contrast, the CBC protocol is less secure than the RBC protocol but has lower complexity, although it cannot provide integrity.

1. Reliable Broadcast Protocol (RBC)

The RBC protocol aims to enable a node to broadcast a message to all other nodes, ensuring that all nodes receive and deliver the message. Its main properties—validity, consistency, integrity, and correctness—are described in detail below (where *m_i_* represents a message *m* with sequence number *i*).

- **Validity:** If an honest node broadcasts a message *m_i_*, then all honest nodes will deliver *m_i_*.
- **Consistency**: If an honest node delivers a message *m_i_* and another node delivers a message *m*′, then *m_i_* = *m*′.
- **Completeness**: If an honest node delivers *m_i_*, then all correct nodes will also deliver *m_i_*.
- **Correctness**: Each honest node delivers *m* only once, and *m* must have been previously broadcast.

RBC was first proposed by Bracha [36], and its implementation involves nodes broadcasting messages to other nodes, which are then rebroadcast by the receiving nodes. Considering the message size *m* in a network of n nodes, the communication complexity reaches $O\left(n^{2}\left|m\right|\right).$ Such a high-complexity broadcasting method is inherently unsuitable for large-scale systems. In 2005, Cachin [37] introduced the use of Merkle trees and corrective censoring techniques, reducing the communication complexity to $O(n\left|m\right|+\lambda n^{2}logn)$. In the (n−2f,2f) corrective censoring code scheme proposed by Cachin et al., the message m is encoded into n fragments and sent to different nodes. Each node uses the received fragment as a leaf node to construct a Merkle tree, enabling verification of the correctness of the received encoding.

2. Consistent Broadcasting Protocol (CBC)

The CBC protocol can be considered a weaker version of the RBC protocol. It only facilitates information transmission between the broadcasting node and the receiving node. The message complexity and communication complexity of the CBC protocol are linear. However, this efficiency comes at the cost of sacrificing integrity. In the CBC protocol, the receiving node relies on the broadcasting node to determine whether a message is delivered. If the broadcasting node is malicious, it can choose not to send a completion message to an honest node, preventing the honest node from delivering the message.

The CBC protocol provides validity, consistency, and correctness. Its consistency and correctness properties are the same as those in the RBC protocol. The validity property is defined as follows:

- **Validity**: If an honest node broadcasts a message *m_i_*, then that node will eventually deliver *m_i_*.

#### 2.2.4. Byzantine Consensus Protocol

Byzantine consensus protocols can be categorized based on their input and output requirements. The next section introduces two commonly used Byzantine consensus protocols: the Asynchronous Binary Consensus Protocol (ABA) and the Verifiable Multi-Valued Byzantine Consensus Protocol (MVBA).

1. Asynchronous Binary Consensus Protocol (ABA)

The ABA protocol requires that both the input and output of nodes are binary values (0 or 1). Although the ABA protocol cannot be directly applied to consensus, it serves as an essential component of Byzantine consensus protocols. The ABA protocol guarantees four key properties: validity, consistency, termination, and correctness. These properties are explained in detail below:

- **Validity**: If all honest nodes input *v*, then any honest node outputs *v*.
- **Consistency**: If an honest node outputs *v*, then all honest nodes output *v*.
- **Termination**: If all honest nodes have inputs, then every honest node will eventually output some value.
- **Correctness**: Honest nodes do not produce duplicate outputs.

The algorithm determines the set of input bits through two or more rounds of message interaction after n nodes receive their input bits. Using a common coin [38], the algorithm randomly decides whether a node outputs its input bit. If not, it modifies the input bit and repeats the process.

2. Verifiable Multi-Valued Byzantine Consensus (MVBA)

The MVBA protocol extends the input space from a binary (0/1) value to any arbitrary value, distinguishing it from other Byzantine consensus protocols through the use of external verification. Honest nodes determine whether to accept or ignore a message based on external validation. The flexibility of the MVBA protocol lies in its ability to adapt through adjustable external validation mechanisms. The MVBA protocol ensures external validation correctness, consistency, and integrity, as described below:

- **External Validation Correctness**: Every honest node will eventually output a message that passes external validation.
- **Consistency**: All honest nodes will output the same message at a specific point in time.
- **Completeness**: If an honest node outputs a value *v*, there must be at least one node that submitted *v* for external verification.

3. Asynchronous Common Subset Protocol (ACS)

The ACS protocol allows each node to input a collection of information and eventually output the collection of all nodes’ input, making it a specialized type of Byzantine protocol. The ACS protocol guarantees validity, consistency, and completeness, which are explained in detail below:

- **Validity**: If a correct node delivers a set *V*, then *V* must contain at least *n* − *f* values, originating from at least *n* − 2*f* honest nodes.
- **Consistency**: If an honest node delivers the information set *V*, then all other honest nodes will deliver the same *V*.
- **Completeness**: If at least *n* − *f* honest nodes provide input, all honest nodes will eventually output a collection of the input information.

## 3. Two-Layer Consensus for Large-Scale IoT

### 3.1. System Model

Blockchain provides reliable, distributed authentication and authorization for IoT devices. IoT data can be stored as immutable and distributed records within the blockchain over time. System participants can verify data authenticity, ensuring that it has not been tampered with. Additionally, blockchain technology enables traceability and accountability for sensor data. Information and communications stored as blockchain transactions are securely protected.

To address scalability challenges caused by the vast number of nodes in large-scale IoT architectures integrated with blockchain, this paper proposes a two-layer scalable framework combining permissioned and permissionless blockchains to support fine-grained transactions in wireless networks. In large-scale industrial IoT systems, message latency and workload can vary significantly among nodes, as each node represents an entity (e.g., a company or organization) involved in maintaining the distributed ledger. To address workload heterogeneity, the proposed architecture uses a permissioned blockchain at the first layer.

**Figure 2:**
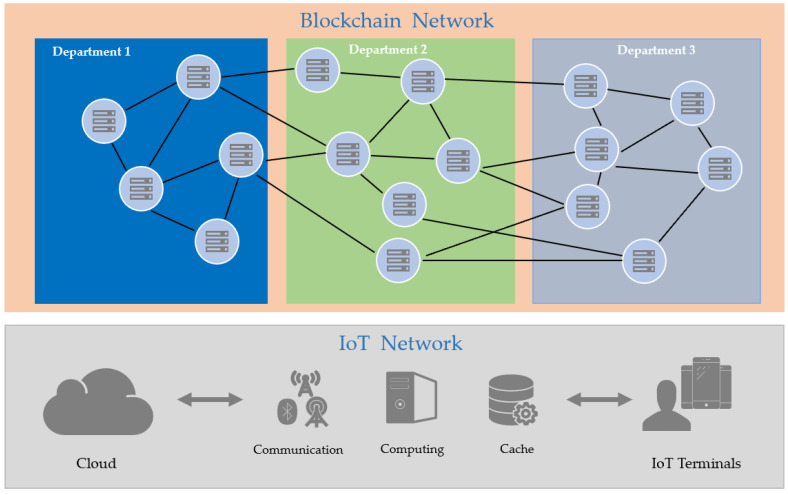
Schematic diagram of the system model.

In industrial parks, each department is equipped with numerous IoT devices, supported by high-performance servers for central control. Each server, acting as a computing node, collects and processes data gathered by the IoT devices within its department. IoT devices collect data, append a timestamp, and send it to the computing devices that represent them. These computing devices sign the data with their private keys on behalf of the IoT devices and then record the signed data on the blockchain. Since the IoT devices within each department are largely identical, their workload is assumed to be balanced. Consequently, a permissionless blockchain is used to achieve consensus among the devices within each department. Given the limited computational and storage resources of IoT devices, only the servers within the department’s central control participate in the consensus process. The system model is illustrated in Figure 2.

The figure above shows the proposed general framework, including the on-chain blockchain system running on the top layer and the IoT wireless network running on the bottom layer. IoT devices are classified into two types of roles, i.e., endorser and terminal. Endorsers are the fixed IoT devices in charge of making consensus and maintaining global consistency, whereas terminals refer to dynamic participating devices that only propose sharing transactions. Both roles have access to communication, computing, and storage resources.

The blockchain network is divided according to the department that the nodes belong to. During a consensus round, the leader of the endorsers organizes the shared transactions in order and forwards them to other endorsers. Upon receipt, all endorsers utilize the selected consensus protocol to reach an agreement collectively. Finally, the endorsers send a reply to the requesting client. Notably, a client can be elected as a new endorser by undergoing authentication and a location check within specific areas. If the current endorsers exhibit abnormal behavior, such as missing blocks or creating forks, they can be removed from the endorser list in the subsequent epoch   t+1.

### 3.2. Two-Layer Chain Design

This paper presents a dual-chain blockchain framework designed to enable fine-grained transactions in large-scale IoT environments. In large-scale industrial IoT, variations in message latency and workload across nodes are common, as each node represents an entity (e.g., a company or organization) responsible for maintaining the distributed ledger.

In industrial parks, each department is equipped with numerous IoT devices, supported by high-performance servers deployed in the department's central control. Each server functions as a computing node that collects and processes data gathered by the IoT devices within its department. IoT devices collect data, append a timestamp, and transmit them to the computing devices that represent them. These computing devices sign the data with their private keys on behalf of the IoT devices, and the signed data are subsequently recorded on the blockchain.

**Figure 3:**
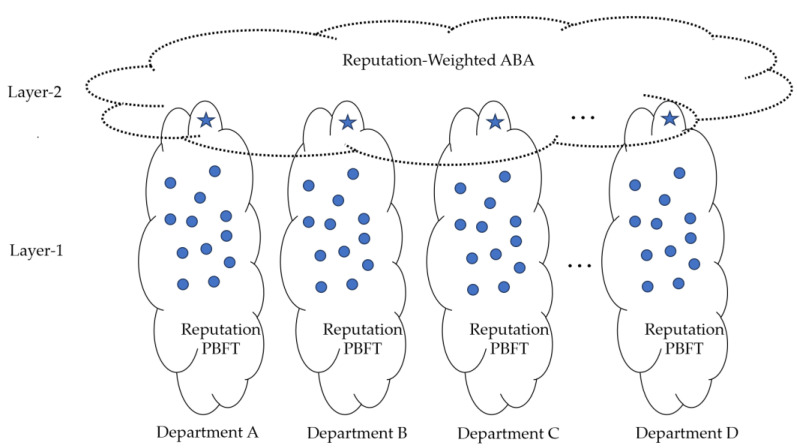
Schematic diagram of Reputation-Weighted Asynchronous BFT.

Blockchain networks are characterized by high computational complexity, limited scalability, significant bandwidth overhead, and latency, which make them unsuitable for resource-constrained IoT nodes. Certain low-power devices are unable to meet the computational demands required for node selection and consensus. A lightweight blockchain consensus mechanism is urgently needed to reduce algorithmic complexity, as well as computational and communication overhead during the consensus process, enabling the inclusion of more lightweight IoT devices. Moreover, security must be ensured during the consensus process to prevent exploitation by malicious actors. Consequently, a reputation-based consensus algorithm is employed to establish consensus among the devices within each department. The system model is illustrated in the Figure 3.

Each dot in the figure represents a node, with nodes enclosed by the same black solid line belonging to the same department. The label “Reputation PBFT” within the circle indicates that the first-layer consensus employs the PBFT algorithm enhanced by a reputation value mechanism. Star-shaped nodes denote group leaders, and for clarity in the figure, group leaders are positioned at the top. Nodes encircled by the gray dotted line represent participants in the second-layer consensus, where only the group leader from each department takes part.

The consensus algorithm in the system involves a total of n participants, which we will refer to as “nodes”, representing the IoT devices engaged in the consensus process. Each node is identified as $\left\{P_{1},\;P_{2},...,P_{n}\right\}$, and each node stores the reputation values of all other nodes. When employing a static Byzantine fault tolerance model, an adversary can corrupt up to f nodes before the protocol begins, where f satisfies the condition 3f+1 ≤ n. These adversary-controlled malicious nodes can engage in arbitrary malicious behavior, collaborating with each other to prevent honest nodes from reaching consensus or to force them into reaching an incorrect consensus. It is assumed that the communication channels between IoT devices are secure. The first layer of the system functions in a weakly synchronous network environment, which accommodates occasional fluctuations in message delivery times.

### 3.3. Layer-1: Reputation-Weighted BFT

The core idea of the algorithm is to evaluate the reputation of all network nodes, conduct voting to elect consensus nodes, evenly group the consensus nodes, and monitor their status. By utilizing intra-group consensus among different groups and global consensus between the main nodes, the algorithm reduces the communication complexity of the traditional PBFT algorithm, thereby improving consensus efficiency.

#### 3.3.1. Reputation Mechanism 

Layer-1 consensus is mainly used to eliminate malicious nodes. To better assess node reliability, reputation values are categorized into different levels. The reputation values of all network nodes are primarily divided into the following four categories, shown in Table 1.

- Excellent Nodes: These nodes actively participate in voting, generate valid blocks, or positively engage in consensus, with their reputation values falling within the range of (*b*,*c*].
- Good Nodes: These nodes actively participate in voting, generate valid blocks, or engage positively in consensus, with their reputation values falling within the range of (*a*,*b*].
- Average Nodes: These nodes have not generated invalid blocks and have not failed to respond during the PBFT consensus process.
- Malicious Nodes: These nodes have failed to actively participate in voting, have generated invalid blocks, or have failed to respond during the PBFT consensus process.

Malicious nodes are disqualified from participating in voting and consensus until the restriction is lifted. Once restored to initial node status, they may rejoin the voting and consensus process.

Reputation value is a crucial metric for assessing a node's reliability within a blockchain network. It is determined by the node's historical consensus performance and its initial configuration. Factors such as network bandwidth, memory, and processor settings during the node initialization process all influence the initial reputation value. The initial reputation value typically ranges between 60 and 75, directly reflecting the quality of server configuration and network environment. Therefore, superior server configurations and favorable network conditions translate into higher initial reputation values for the nodes. These reputation values are periodically updated throughout the continuous consensus process. Generally, the primary factor for grouping nodes is their respective reputation values. Nodes with higher reputation values are identified as non-Byzantine nodes and are subsequently assigned to the high-reputation group. In contrast, nodes with relatively lower reputation values are classified as Byzantine nodes and are assigned to the backup node group. 

The proposed reputation value mechanism aims to eliminate Byzantine nodes as effectively as possible, select highly reliable consensus nodes, and enhance the security of the consensus process. The model evaluates the behavior of nodes participating in voting and consensus, and after each round of consensus, it calculates the reputation values of the nodes and classifies them into reputation levels. This classification helps in identifying Byzantine nodes, enabling the system to accurately detect and eliminate malicious nodes. To prevent the risk of centralization due to excessively high reputation values, a voting mechanism is introduced. Nodes can vote for the nodes they trust, meaning that the selection of consensus nodes is not solely based on reputation values but also on the number of votes each node receives. After the voting process concludes, the system will tally the votes and reputation values of the elected nodes. The calculation formula is as follows:

(1) R=P∗α+C∗β

where R represents the calculated result, P represents the number of votes received by the elected node, and C represents the reputation value. α and β(α+β=1) represent the weights assigned to the number of votes and the reputation value, respectively.

To accurately capture node behavior logs, logging points are inserted during the election and consensus processes. The interactions and feedback between nodes are used as evaluation criteria. If a node fails to provide feedback or provides incorrect information, its behavior is considered abnormal, resulting in a deduction of the corresponding reputation value. If the node provides correct feedback, its behavior is deemed positive, and its reputation value is increased.

(2) $$C=C-\sum_{i=1}^{n}p_{i}+\sum_{i=1}^{n}o_{i}$$

where C represents the reputation value, $\;p_{i}$ denotes the deduction in reputation value for abnormal node behavior, and $o_{i}$ denotes the reward in reputation value for positive node behavior. The reputation values of nodes participating in elections and consensus across the network are dynamic. Nodes that actively participate in the voting process see their reputation values increase, while non-malicious nodes that do not participate in voting experience a decrease in their reputation values. After a round of voting, nodes that join the consensus group participate in the block generation and validation process. Consensus nodes that successfully generate a block will have their reputation values increased. Nodes that fail are removed from the consensus group and prohibited from participating in the next voting round for a specified period of time, t. Nodes with reputation values below a are prohibited from participating in elections or being elected until the restriction is lifted, after which they return to their initial node status. Nodes that reach the maximum reputation value c and have a block generation record are reset to their initial node status.

**Figure 4:**
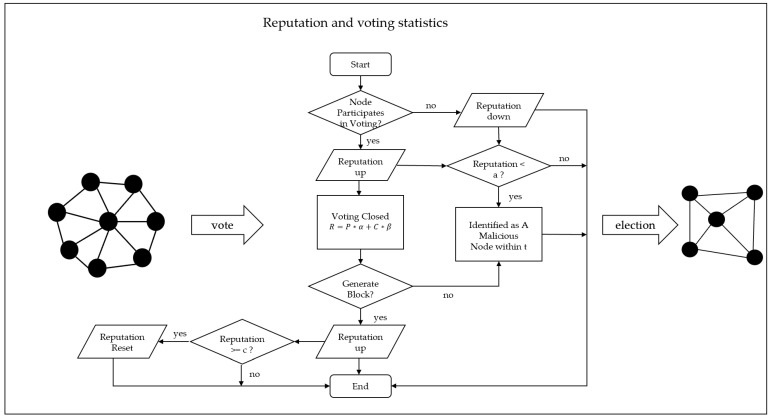
Consensus Node Election Model Diagram.

Newly added nodes are assigned an initial reputation value of a, referred to as initial nodes. During the first cycle of the consortium blockchain’s operation, the result of the first voting round depends solely on the number of votes each node receives. The selection of consensus nodes is determined by the number of votes each node obtains. In each operational cycle, malicious nodes are excluded from the voting process. The final election results are determined based on the number of votes, reputation values, and their respective weights assigned to the elected nodes. The schematic diagram of the process of nodes voting based on reputation value is shown in Figure 4.

In the consortium blockchain network, all non-malicious nodes are referred to as ordinary nodes, and each ordinary node can vote for nodes it trusts. After the voting process is completed, the system tallies the votes and reputation values of each node. It ranks the nodes using the reputation voting model and selects consensus nodes to form the consensus group. In response to irregular or inactive behavior during the voting process, the reputation evaluation mechanism adjusts the reputation values of the nodes accordingly. The inclusion of the reputation voting mechanism helps reduce malicious node behavior and encourages active participation in the voting and consensus processes. This mechanism reduces the likelihood of Byzantine nodes being reselected as consensus nodes. It also mitigates the centralization risk caused by excessively high reputation values, thereby improving consensus security effectively.

#### 3.3.2. Weight Calculation

As mentioned earlier, the first layer of consensus is mainly used for ensuring data consistency among devices within a department, meaning that natural grouping has already been established. Thus, there is no need to introduce additional grouping algorithms, which would only increase the complexity of the process.

After the reputation value voting algorithm is executed, the groups in each department reach a consensus and form a unified group. The $P_{i}$ node obtains all group information and identifies the leader node of each department. Once a timer is set, $P_{i}$ sends the information to all nodes, notifying all group leaders to calculate the reputation weight.

After receiving the message, the group leader will first verify the signature in the message. If the verification fails, the message will be ignored. If the verification is successful, the weight calculation will be divided into the following two cases:

1. Group leaders who receive the corresponding REPLY messages (including their own REPLY messages, if applicable) invoke the aggregation function to combine the signatures of $<H\left(mi\right),1>$ in all received REPLY messages. If a group leader receives w1 REPLY messages, the leader’s weight is w1. This weight is directly influenced by the number of group leaders participating in the group announcement, as only nodes with sufficiently high reputation (not removed from the current consensus round) will send messages. Along with the aggregated signature, the group leader must also provide the relevant ID set and pk set within the aggregated signature, enabling other group leaders to verify the weight.
2. Group leaders who have not received the corresponding REPLY message broadcast the NO_REPLY message to the nodes in the group, start a timer, and set an appropriate duration. Upon receiving the NO_REPLY message, node $P_{j}$ in the group verifies whether it has received a block m that satisfies the condition $H(m)=H(m_{i})$. If it is confirmed that no block satisfying the condition has been received, $P_{j}$ sends a NO_BLOCK message to its group leader, where <H(mi),0> is included in the NO_ BLOCK message. The value 0 represents the input for the subsequent RWABA algorithm. Once the timer set by the group leader expires, the group leader aggregates the signatures of <H(mi),0> from all received NO_BLOCK messages. Therefore, if the group leader receives w2  NO_BLOCK messages, the leader’s weight is w2.

### 3.4. Layer-2: RWBFT

#### 3.4.1. RWABA

In the first layer of the consensus algorithm, as nodes within a group are located in the same department, group communication is stable, making the PBFT weakly synchronous consensus algorithm suitable. A reputation-based mechanism is introduced to enhance consensus efficiency. However, since the leader nodes of different departments are physically more distant, communication between groups is more prone to external interference, leading to significantly larger fluctuations in message delivery delays between group leaders compared to intra-group communication. If the PBFT algorithm is used between group leaders (in the second layer of consensus), there is a high chance that the election of a new primary node will fail during the view change phase due to delayed message delivery when a leader node fails. Once the election of the new primary node fails, the node’s timeout timer doubles. The timeout period for PBFT’s timer only increases and never decreases, causing all subsequent processes to have extended timeout periods, which negatively impacts the efficiency of all future consensus operations.

Attention is, therefore, directed toward asynchronous consensus algorithms. In the application scenario, nodes within each group are ranked based on their reputation values, and nodes with lower reputation values are excluded. The more nodes that have reached the first layer of consensus within a group, the higher the number of high-reputation nodes, giving the group greater ability to drive the second layer of consensus. The term “reputation-weight” is used to represent the contribution of a group to achieving the second layer of consensus. In the second-layer consensus process, there are m participants, corresponding to the number of departments, which are referred to as “nodes”. Each node is represented by the set $\left\{P_{1},P_{2},P_{3},...,P_{m},\right\}$, and each is assigned a reputation-weight $\left\{{rw}_{1},{rw}_{2},{rw}_{3},...,{rw}_{m},\right\}$ based on its ability to contribute to the consensus. This mechanism is analogous to holding different amounts of currency in Proof of Stake (PoS). 

Since the FLP impossibility theorem rigorously proves that no deterministic algorithm can achieve consensus in an asynchronous network, a common coin is introduced as a globally visible entity. All nodes can access the same random bit string $b_{1},b_{2},\ldots ,b_{r},\ldots$ generated by the common coin. Each bit $b_{r}$ can only take a value of 0 or 1, with an equal probability of 50% for both values. 

Assuming that the block corresponding to the current consensus is m, and r represents the number of rounds of RWABA, the hash function is applied to perform a hash operation on the hash value of the message and the number of rounds, i.e., H(H(m)|r)), to introduce randomization. The irreversibility of the hash function prevents malicious nodes from constructing a desired block based on the output and ensures that the probability of 0 and 1 in the output is uniformly distributed. Algorithm 1 is a Reputation-Weighted Asynchronous binary Byzantine fault-tolerant algorithm.
**Algorithm 1.** Layer-2: RWABA**Initialization:** $\mathrm{When}\;\mathrm{node}\;P_{i}$ $\mathrm{receives}\;\mathrm{the}\;\mathrm{input}{\;b}_{input}$ $,\;\mathrm{set}\;{{est}_{0}\;:=b}_{input},\;\;decided\;:=false,$ r:=0 $,\;{bin\_values}_{r}:=\emptyset$ $\mathrm{and}\;\;{RWeightSum}_{r}^{d}\;:=0$, where r={0, 1, 2,...} and d={0, 1}.1:${RWeightSum}_{r}^{{est}_{r}}\;:={rw}_{input}$;2:$\mathrm{broadcast}\;\mathrm{to}\;\mathrm{other}\;\mathrm{nodes}\;\;{Val}_{r}\left({est}_{r},rw_{i}\right)=\;\{Val,r,{est}_{r},rw_{input}\}$3:**Upon** 
$\mathrm{receiving}\;{Val}_{r}\left(v,rw\right)$
$\;\mathrm{from}\;P_{j}\;\;$
**do**
4:        ${RWeightSum}_{r}^{v}\;:={RWeightSum}_{r}^{v}+rw$;5:**Upon** 
${RWeightSum}_{r}^{v}\geq f+1\;\;$
**do**
6:        If ${Val}_{r}\left(v,rw_{input}\right)$ has not been sent, then broadcast ${Val}_{r}\left(v,rw_{input}\right)$.7:**Upon** 
${RWeightSum}_{r}^{v}\geq 2f+1\;\;$
**do**
8:        ${bin\_values}_{r}\;:={bin\_values}_{r}\cup \{v\}$;9:**Upon** 
${bin\_values}_{r}\neq \emptyset \;$
**do**
10:        
$broadcast\;{AUX}_{r}\left(\mu ,{rw}_{input}\right)=\{AUX,r,\mu ,rw_{input}\}$
$,\;\mathrm{where}\;\mu \in {bin\_values}_{r}$
11:        $\mathrm{Wait}\;\mathrm{until}\;\mathrm{the}\;\mathrm{sum}\;\mathrm{of}\;\mathrm{the}\;\mathrm{reputation}\;\mathrm{weights}\;\mathrm{in}\;{AUX}_{r}\left(\mu ,*\right)$ is greater than or equal to n−f$,\;\mathrm{then}\;\mathrm{set}\;\;{val}_{r}$ as the set of x $\mathrm{in}\;\mathrm{the}\;{AUX}_{r}$$,\;\mathrm{resulting}\;\mathrm{in}\;{val}_{r}\subseteq {bin\_values}_{r}$.12:        s :=H(H(m)|r);13:        
**if** 
${val}_{r}=\{b\}$ 
**then**
14:              
**if** 
b=s % 2 
**then**
15:                      
**if** 
decided=false 
**then**
16:                              reach consensus b;17:                              
decided≔true
*;*
18:                      
**else:**
19:                              terminate program;20:              ${est}_{r+1}≔b$;21:        
**else:**
22:              ${est}_{r+1}≔s\%2$;23:r≔r+1;24:goto line 1;

The output b of the RWABA algorithm determines whether all nodes accept the corresponding block (b=0 indicates discard, while b=1 indicates accept). The leader of each department participating in the second-layer consensus forwards the RWABA algorithm’s output to the department members. If b=1, department members retain the corresponding block and sort the blocks by their timestamps; otherwise, the block is discarded.

The algorithm runs in a loop based on rounds, exiting only when the conditions are met. The local variable ${est}_{r}$ represents the node’s current “estimated value”, which is the candidate value expected to reach consensus, while r represents the current round number. The operations of node $P_{i}$ in round r can be roughly divided into the following three parts:

- **Phase 1:** Lines 1–8, in which the primary task is exchanging and propagating the estimated values. Node $P_{i}$ first adds its own weight to the local “weight sum” variable ${RWeightSum}_{r}^{{est}_{r}}$ and broadcasts a message ${Val}_{r}({est}_{r},rw_{input})$, which contains its current estimated value and weight, to all other nodes. Upon receiving a ${Val}_{r}(v,rw)$ message from other nodes, the weight from the message is added to the corresponding ${RWeightSum}_{r}^{v}$. Once the weight for a particular estimated value exceeds f+1, it proves that at least one honest node has sent the message (since the adversary’s control cannot exceed f, and if no corresponding message was sent earlier, the adversary can follow by sending the same estimated value along with its own reputation-weight). When the weight of a particular estimated value exceeds 2f+1, the value is considered to have been sufficiently exchanged, and it is placed into the set ${bin\_values}_{r}$, allowing the algorithm to proceed to the next phase.
- **Phase 2:** Lines 9-11: The main task in this phase is still the exchange and dissemination of information. Once a node finds that its local set ${bin\_values}_{r}$ is not empty, it will broadcast the message ${AUX}_{r}(\mu ,{rw}_{input})$, informing other nodes that the estimated value μ has entered the second phase. As in the first phase, when the node receives enough messages such that the reputation-weight of the estimated value entering the second phase exceeds n−f, it can be assumed that enough honest nodes have taken this estimated value as a candidate for consensus, and the node can proceed to the third phase.
- **Phase 3:** Lines 12–23, where the primary task is to reach the final consensus. At the beginning of the third phase, it is confirmed that a sufficient number of honest nodes are ready to reach a consistent consensus result, which corresponds to their local estimated value. In this phase, a common coin is introduced to bring in randomization, which is crucial for allowing the protocol to function in an asynchronous network. When the common coin’s value aligns with the local estimated value, the local value can be adopted as the final consensus result. If the values do not match, all honest nodes’ estimated values are unified, and the process continues to the next round. Once in the third phase, the probability of reaching final consensus in each round is 50%. As the number of rounds increases, the probability of remaining stuck in the loop decreases exponentially, leading to the conclusion that the expected number of rounds for the algorithm is constant.

#### 3.4.2. Safety and Feasibility Analysis

The proposed protocol must satisfy the following four properties to ensure the security and reliability of the consensus algorithm:

- **Validity**: The consensus result ultimately obtained by honest nodes is guaranteed to originate from inputs provided by honest nodes.
- **Consistency**: All honest nodes will eventually agree on the same consensus result.
- **Conclusion Singleness**: In each round of consensus, honest nodes will only reach a single consensus decision.
- **Terminability**: All honest nodes will eventually reach a consensus, ensuring the process does not remain perpetually unresolved.

**Correctness**: Honest nodes do not produce duplicate outputs.

Next, a proof is provided to demonstrate that RWABA satisfies the four properties mentioned above. First, several symbolic definitions are introduced. n represents the sum of the weights of all nodes, f represents the sum of the weights of malicious nodes controlled by the adversary, and f satisfies 3f+1≤n. r denotes a specific cycle in the RWABA algorithm.

**Lemma 1.**  *If the weights of honest nodes that use the same input value v sum to more than f* + 1*, then v will eventually be added to the set of bin_values of all honest nodes*.

**Proof.**  In a reliable peer-to-peer communication network, every honest node eventually receives the message *v* with a weight sum exceeding *f* + 1. As a result, the condition in line 5 is satisfied, prompting each honest node to broadcast a *val*. Given that the adversary’s control weight is at most *f*, the weight sum of *v* eventually reaches or exceeds 2*f* + 1, satisfying the condition in line 7. Thus, *v* is added to the *bin_values* set of all honest nodes, and subsequently included in their local *bin_values* collections. □

**Lemma 2.**  *If a node* $P_{i}$ *is honest and* v∈bin_values*, then at least one honest node must have used* v *as an input*.

**Proof.**  This lemma is equivalent to stating that a message v, sent exclusively by malicious nodes, will never be added to the bin_values set of any honest node. Assume that only malicious nodes send the message v. Since the weights controlled by malicious nodes are not sufficient, v cannot be added to the bin_values collection. As malicious nodes control at most f weight, the honest nodes’ combined weights for v are limited to f. The conditions in lines 5 and 7 are never satisfied, ensuring that the message v sent by malicious nodes is never added to the bin_values set of any honest node. □

- Third bullet.

**Lemma 3.**  *If v is added to the bin_values set of an honest node P_i_, then eventually all other honest nodes will include v in their bin_values sets*.

**Proof.**  If v  is included in the bin_values set of an honest node $P_{i}$, then $P_{i}$ must have received vvv such that the weight sum of the messages received by $P_{i}$ exceeds 2f+1. As the adversary controls at most f weight, the cumulative weight of the messages supporting v sent to $P_{i}$ exceeds f+1. Since the adversary's control weight does not exceed f, the total weight of nodes sending the message containing v cumulatively exceeds f+1f. Therefore, the condition in line 5 is eventually satisfied, prompting all honest nodes to broadcast the message containing v. Consequently, the condition in line 7 is also satisfied (as n−f≥2f+1), ensuring that all other honest nodes add v to their bin_values collections. □

**Lemma 4.**  *Since all honest nodes provide inputs when participating in the consensus, the* bin_values *set of every honest node will eventually be non-empty*.

**Proof.**  The following conditions hold: (a) The total weight of honest nodes is at least n−f. (b) Every honest node provides input to the consensus. (c) $n-f\geq 2f+1=\left(f+1\right)+f$. (d) Honest nodes only input valid values into the consensus. Thus, it can be inferred that at least one of the values 0 or 1 has sufficient weight. Combining this with Lemma 1, it follows that the bin_values set of all honest nodes will eventually be non-empty.

By combining Lemma 1 through Lemma 4, we conclude that if all honest nodes participate in the consensus, the bin_values set is guaranteed to be non-empty. The bin_values set contains all messages broadcast by honest nodes and excludes messages broadcast exclusively by malicious nodes. □

## 4. Experiment Results

An asynchronous dual-layer Byzantine Fault Tolerance (BFT) algorithm is proposed for large-scale IoT blockchain systems. The first layer incorporates a reputation mechanism to eliminate malicious nodes, while the second layer employs asynchronous consensus to significantly enhance the scalability of the consensus algorithm. The experiment is run in a personal computer environment. The specific experimental environment configuration details are shown in the Table 2 below.

The main experiment was conducted on a laptop equipped with an Intel(R) Core(TM) Ultra 5 125H CPU running at 1.20 GHz and 32 GB of memory, with the consensus processes simulated using Go. The experiment focused on simulating and analyzing the throughput and latency of the proposed consensus algorithm. Subsequently, the proportion of malicious nodes was adjusted to observe how the consensus latency of the hybrid dual-layer Byzantine Fault Tolerance (BFT) algorithm responded under varying numbers of malicious participants. This analysis was aimed at demonstrating that our consensus algorithm maintains strong performance, even in the presence of malicious nodes. 

The RWA-BFT research prototype was evaluated against two baselines. First, it was compared to an independently implemented version of PBFT, which is currently the most widely used consensus algorithm in the IoT field and serves as a classic BFT protocol for partially synchronous networks. This comparison highlights the advantages of asynchronous protocols. Second, RWA-BFT was compared to HB-BFT, the first protocol of the new generation of asynchronous BFTs. To obtain an apples-to-apples comparison, the unmodified HB-BFT code base was deployed, and efforts were made to optimize its implementation as much as possible.

### 4.1. Throughput

System throughput refers to the number of transactions processed by the system per unit of time, making it one of the most direct indicators for evaluating the performance of a consensus algorithm. In the throughput experiment, a blockchain environment with different consensus mechanisms was simulated using the Go programming language. The experiment used 100,000 real Ethereum transaction data points, with the monitoring node configured to send 2000 transactions per second. In the experiment, the average TPS values of PBFT and the reputation-based PBFT improvement proposed in this paper were compared, taking into account varying numbers of nodes within each group.

**Figure 5:**
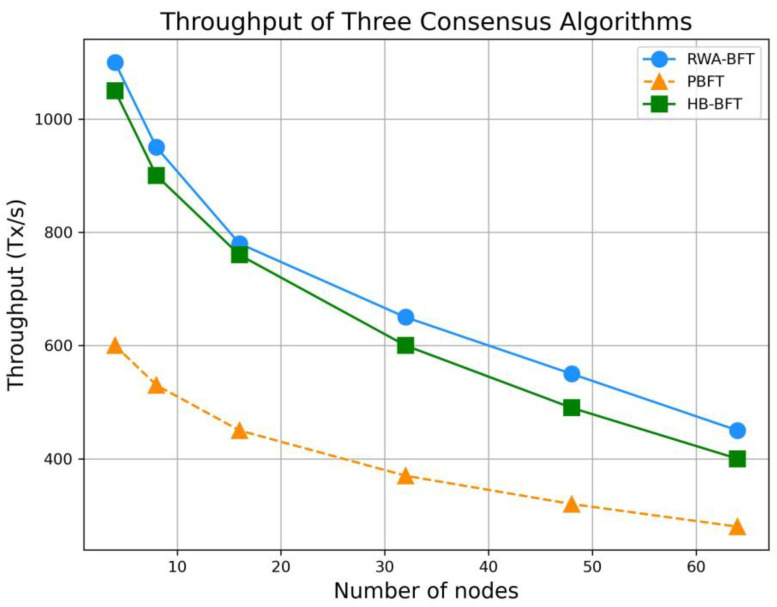
Throughput comparison between RWA-BFT, PBFT, and HB-BFT.

Figure 5 above shows that the throughput of three consensus algorithms decreases as the number of nodes increases. Since the first-layer consensus algorithm proposed in this paper eliminates low-reputation nodes, it reduces the number of nodes involved in the consensus, optimizing the overall consensus process. Therefore, under the same number of consensus nodes, the throughput of consensus proposed in this paper will always be higher than that of the PBFT algorithm and HB-BFT algorithm.

The comparison between RWA-BFT and HB-BFT in the figure above shows that when the number of nodes is small, the throughput of the two protocols is similar. This is because the critical path of ABA remains the same for both protocols under normal conditions. However, as the number of nodes increases, the throughput gap between RWA-BFT and HB-BFT widens. This is attributed to RWA-BFT’s ability to increasingly screen out malicious nodes through the reputation value mechanism in the first layer.

### 4.2. Latency

We start timing when a block is broadcast and stop once the block is confirmed on the blockchain. The time elapsed during this period represents the consensus algorithm’s latency. Then, we gradually increase the number of nodes from 4 to 500, observing and recording the changes in latency for three consensus algorithms.

**Figure 6:**
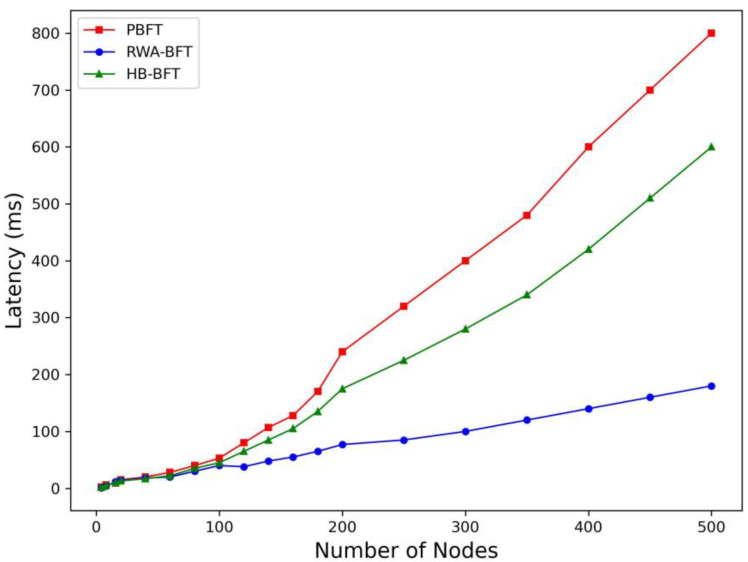
Comparison of the latency between RWA-BFT, PBFT, and HB-BFT when the number of nodes continues to increase.

The latency comparison of RWA-BFT, PBFT, and HB-BFT when the number of nodes continues to increase is shown in Figure 6. As shown in the experimental results, the latency of RWA-BFT consistently remains low. This is because, despite the increasing total number of nodes, the reputation value mechanism reduces the number of nodes participating in the consensus.

By examining the experimental results’ curve for the RWA-BFT protocol, it can be observed that when the number of nodes reaches 100, there is a noticeable fluctuation in consensus latency, which significantly exceeds the latency at 120 nodes. For the rest of the curve, the latency increases monotonically as the number of nodes increases. This is due to the RWABA algorithm, which is introduced in the second layer of the protocol’s consensus mechanism and includes randomization. While the expected number of rounds remains constant, the actual number of rounds executed can vary. In the actual experiment, when the number of nodes reached 100, the RWABA rounds significantly exceeded the expected number, which caused the fluctuation seen around 100 nodes in the experimental graph. This situation can be alleviated or avoided by increasing the number of experiments with the same number of nodes.

### 4.3. The Impact of the Proportion of Malicious Nodes

This part of the experiment focuses on verifying the robustness of the RWA-BFT algorithm. To simulate a large-scale IoT environment, we introduced varying proportions of malicious nodes into the original experimental setup to evaluate their impact on the RWA-BFT, PBFT, and HB-BFT consensus. Additionally, network delay and packet loss were simulated within the container using the Linux Traffic Control tool.

**Figure 7:**
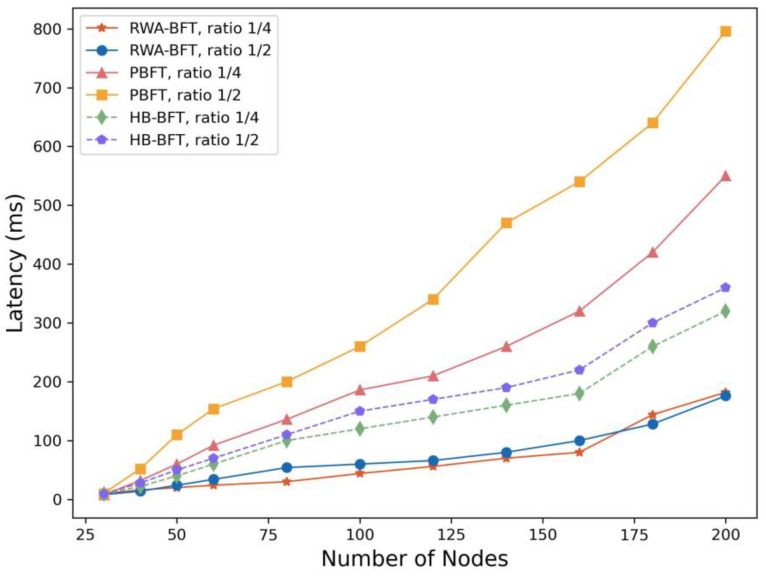
Comparison of the latency between RWA-BFT, PBFT, and HB-BFT when the number of nodes continues to increase under different proportions of malicious nodes.

The experimental results is shown in Figure 7. The figure indicates that when malicious nodes account for 1/4 and 1/2, the delay of PBFT increases significantly with the growing number of nodes, and the rate of increase accelerates. In contrast, HB-BFT, as the first asynchronous consensus algorithm, exhibits higher latency but does not display a significant upward trend as the proportion of malicious nodes rises. Meanwhile, the RWA-BFT algorithm shows a slower growth rate in latency, with its two curves alternating in increase. These results demonstrate that asynchronous consensus algorithms offer better robustness than semi-synchronous consensus algorithms and are more suitable for IoT scenarios.

## 5. Conclusions

This paper proposes a dual-layer consensus mechanism for blockchain in large-scale IoT systems. In the first layer, a reputation-based mechanism filters out low-reputation nodes within departments, reducing the number of nodes participating in consensus. Additionally, considering specific IoT scenarios, nodes from the same department are treated as a group, eliminating the overhead of node grouping. In the second layer, the participants are the highest-reputation nodes from each group in the first layer, and an asynchronous consensus algorithm based on "reputation-weight" is implemented. Experimental results show that the proposed algorithm delivers high throughput and low latency, aligning well with the high scalability demands of blockchain applications in IoT environments. It is worth highlighting that the asynchronous consensus algorithm maintains strong robustness in the complex network environment of large-scale IoT systems, which has practical significance for the integration of blockchain and IoT. 

The dual-layer consensus mechanism shows great potential for practical applications in areas such as smart cities, industrial IoT, healthcare, and supply chain management, where scalability, security, and low latency are essential. Future research directions could include optimizing reputation mechanisms, improving energy efficiency, mitigating advanced security threats, and exploring integration with hybrid blockchain architectures or real-world IoT scenarios to enhance its applicability and robustness.

Due to certain limitations, this protocol still has significant potential for improvement. To adopt blockchain technology in IoT environments, it is essential to address the challenges posed by the limited storage and computing resources of IoT devices. The prevailing solution involves leveraging edge computing, but the storage and computational capabilities of edge devices remain constrained. To mitigate the storage challenges in IoT blockchains, some researchers have proposed using distributed hash tables to enable data storage across all nodes in a distributed manner. Investigating how to seamlessly integrate a highly scalable consensus algorithm optimized for IoT blockchain scenarios with this storage approach represents an important avenue for further research and development.

## Figures and Tables

**Table 1:** Node classification table.

Node Classification	Reputation Value Range
Malicious Node	[0,a)
Initial Node	a
Good Node	(a,b]
Excellent Node	(b,c]

**Table 2:** Experimental environment configuration table.

Configuration Items	Configuration Details
Operating system	Ubuntu 22.04
CPU	Intel(R) Core(TM) Ultra 5 125H 1.20 GHz
Memory size	32 GB
Disk capacity	1TB
Development environment	Visual Studio Code
Golang version	Go1.23.1

## Data Availability

The original contributions presented in this study are included in this paper. Further inquiries can be directed to the corresponding authors.
